# Mechanism of Action and Therapeutic Potential of Sulforaphane in Skeletal Muscle Diseases: Molecular Pathways and Precision Medicine

**DOI:** 10.1002/fsn3.72154

**Published:** 2026-07-22

**Authors:** Minru Zhao, Bo Huang, Juan Chen, Junya Li, Zhongli Zhu, Guoyun Zhu, Jian Hu, Fuxiang Li, Jian Feng

**Affiliations:** ^1^ Department of Clinical Pharmacy The General Hospital of Western Theater Command Chengdu Sichuan China; ^2^ Department of Burn and Plastic Surgery The General Hospital of Western Theater Command Chengdu Sichuan China; ^3^ Department of Critical Care Medicine The General Hospital of Western Theater Command Chengdu Sichuan China

**Keywords:** bioavailability, diabetic myopathy, ICU‐acquired weakness, mitochondrial function, mTOR signaling pathway, nutraceutical, precision medicine, sarcopenia, skeletal muscle, sulforaphane

## Abstract

Sulforaphane (SFN), a bioactive isothiocyanate abundant in cruciferous vegetables, has attracted growing interest as a potential nutraceutical intervention for skeletal muscle disorders. This narrative review synthesizes preclinical and early clinical evidence on the mechanisms and therapeutic applicability of SFN in ICU‐acquired weakness, diabetic myopathy, sarcopenia, and exercise‐induced muscle damage. SFN modulates skeletal muscle pathophysiology through four interconnected axes: regulation of protein homeostasis via mTOR‐associated signaling and suppression of ubiquitin‐proteasome‐mediated catabolism; upregulation of oxidative stress defenses through Nrf2‐driven antioxidant enzyme induction; attenuation of inflammatory networks via NF‐κB inhibition and promotion of M2 macrophage polarization; and metabolic reprogramming through AMPK‐mediated mitochondrial biogenesis and enhanced insulin sensitivity. Preclinical data suggest preliminary protective effects on respiratory and locomotor muscle; however, clinical translation remains uncertain. Several randomized controlled trials have failed to demonstrate significant effects on Nrf2 target genes or metabolic stress biomarkers, and bioavailability varies markedly across formulations and individuals. Heterogeneous responses across muscle fiber types, an undefined therapeutic window, and interindividual variability in gut microbiota‐mediated conversion further complicate clinical application. To address these challenges, we propose a conceptual “Sulforaphane Precision Medicine Framework” integrating molecular biomarkers, gut microbiome profiling, and dynamic delivery systems to guide personalized intervention. While SFN shows promise as an adjunctive therapy, standardized formulations, refined patient stratification, and rigorous phase III trials are essential before routine clinical adoption can be recommended.

## Graphical abstract

Sulforaphane modulates mTOR, Nrf2, NF‐κB, and AMPK/PGC‐1α pathways to protect skeletal muscle and enable precision‐medicine intervention in ICU‐acquired weakness, sarcopenia, and diabetic myopathy.

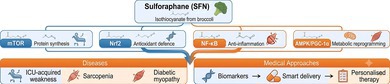

**Abbreviations**
AMPK: AMP‐activated protein kinase
ARE: Antioxidant Response Element
ASD: autism spectrum disorder
COPD: chronic obstructive pulmonary disease
FOXO3a: Forkhead box O3a
GLUT4: glucose transporter type 4
GRAS: Generally Recognized as Safe
GSH: glutathione
GSTM1: glutathione S‐transferase mu 1
GSTs: Glutathione S‐transferases
ICU: (intensive care unit)
ICU‐AW: ICU‐acquired weakness
IκB: inhibitor of NF‐κB
Keap1: Kelch‐like ECH‐associated protein 1
LC3: microtubule‐associated protein 1A/1B‐light chain 3
LD₅₀: median lethal dose
mTOR: mechanistic target of rapamycin
NF‐κB: nuclear factor kappa‐B
Nrf1: nuclear factor erythroid 2‐related factor 1
Nrf2: (nuclear factor erythroid 2‐related factor 2)
PFKFB3: 6‐phosphofructo‐2‐kinase/fructose‐2,6‐bisphosphatase 3
PGC‐1α: peroxisome proliferator‐activated receptor gamma coactivator 1‐alpha
SFN: sulforaphane
SOD: superoxide dismutase
TDM: therapeutic drug monitoring
TFAM: mitochondrial transcription factor A

## 1 Introduction

Skeletal muscle is important for overall health and physiological homeostasis. In addition to its role in locomotion (contractile function), skeletal muscle also participates in other functions such as metabolism and immune response. In addition to being the largest organ by absolute amount (40% of total body mass), skeletal muscle is central to resting energy expenditure and exercise capacity (Huang et al. 2022). Yet, with aging or after serious illness such as ICU‐acquired weakness and sarcopenia, muscle mass and muscle function both undergo considerable loss (Chen et al. 2023). In addition, severe invasive surgical procedures, chronic obstructive pulmonary disease (COPD), and diabetes also lead to skeletal muscle wasting that seriously affects the quality of life and recovery of patients (Tanaka et al. 2020). Current interventions to prevent muscle loss include anabolic steroids, which exploit the endogenous anabolic pathway, but with their side effects being so severe that they are not used clinically (Albano et al. 2021). Rehabilitation therapy is an option too, but that is ineffective in severely ill patients. The lack of effectiveness of both interventions calls for safer and more efficacious interventions.

Given the limitations of available treatments, the severe side effects of anabolic steroids, and the narrow applicability of rehabilitation therapy in critically ill patients, it is important to develop drugs that could be complementary to the existing treatments. Plant‐derived bioactive compounds—for example, the phytochemical sulforaphane (SFN)—offer several potential advantages. From a therapeutic point of view, they often act on multiple targets, induce endogenous protective pathways, and have favorable side effect profiles compared to synthetic drugs. Sulforaphane (SFN) is a phytochemical abundant in cruciferous vegetables (mainly broccoli) that has recently drawn attention as a potential therapeutic agent in skeletal muscle‐related processes (Kaiser et al. 2021; Vanduchova et al. 2019). The bioactivation of glucoraphanin to SFN depends critically on gut microbiome activity (Fahey et al. 2015), which governs systemic bioavailability and may facilitate enrichment in skeletal muscle, indicating potential tissue‐specific bioactivity. Improved understanding of SFN's mechanisms of action (and limitations) is needed to establish whether SFN might have a future adjunct role in treating skeletal muscle disorders.

## 2 Methods

Our narrative review was guided by a systematic search in PubMed/MEDLINE, Web of Science, and Scopus from inception to May 1, 2025, and additionally tracked citations and manually screened reference lists to identify recent peer reviewed articles through May 2026. Our search was based on a combination of Medical Subject Headings (MeSH) terms and free text terms using Boolean operators. Our main search string was: “sulforaphane OR SFN OR glucoraphanin OR broccoli sprout extract” AND “skeletal muscle OR muscle atrophy OR myopathy OR ICU‐acquired weakness OR sarcopenia OR diabetic myopathy OR diaphragm OR respiratory muscle” AND “mTOR OR NF‐κB OR AMPK OR oxidative stress OR inflammation”. Included were original peer reviewed journal articles (in vitro, animal or human trials) and good quality systematic reviews/meta‐analyses reporting any outcome measures related to muscle mass, muscle contractile function, muscle metabolism, and related pathways.

Exclusion criteria were: conference abstracts, editorials, opinion and review articles, case reports not based on original data or lacking original data, and non‐English articles. We included preprints only if no other peer reviewed publication was available, and that information was explicit. We did not include articles that study other isothiocyanates than SFN and are not relevant to SFN, or articles that study tissues other than muscle without any relevance to muscle functioning. We summarized evidence narratively, and differences between preclinical and clinical evidence, as well as between acute and chronic effects of supplementation, were clearly identified.

## 3 Chemical Properties and Biological Activity of SFN

### 3.1 Chemical Structure and Source Analysis

SFN is a bioactive phytochemical belonging to the glucosinolate family, and is a common compound of cruciferous vegetables such as broccoli, cabbage, and radish (Kaiser et al. 2021; Vanduchova et al. 2019). This represents an evolutionary adaptation in which these plants produce defense compounds (glucosinolates, such as glucoraphanin, the precursor of SFN) against insects, pathogens, and other stresses (Kaiser et al. 2021; Vanduchova et al. 2019). Recent evidence indicates that endophytic fungi colonizing Brassica roots can further modify the host glucosinolate profile (Poveda et al. 2022). SFN is an isothiocyanate with a characteristic sulfur atom group (*R* – *N* = C = S). Isothiocyanates produced from the hydrolysis of glucosinolates have various biological activities such as anti‐cancer and anti‐inflammatory activities (Kaiser et al. 2021; Orouji et al. 2023). From a dietary perspective, sprouts, especially broccoli sprouts, are a rich source of SFN in foods. Food processing affects the stability and bioavailability of SFN; high cooking temperatures, such as frying, destroy heat‐labile myrosinase and SFN; and milder food processing methods preserve more myrosinase and SFN (Yagishita et al. 2019).

Dietary intake of sulforaphane through cruciferous vegetables differs markedly from pharmacological doses used in experimental studies. A typical serving of broccoli provides only a fraction of the sulforaphane concentration tested in cell and animal models, whereas standardized supplements can achieve plasma concentrations orders of magnitude higher (Fahey et al. 2015; Yagishita et al. 2019). This discrepancy raises important questions about the translational relevance of preclinical dosing regimens to dietary recommendations and nutraceutical applications.

### 3.2 Sulforaphane Bioavailability: From Nutraceutical Spectrum to Functional Foods

Sulforaphane is generally derived from its prodrug glucoraphanin (GRA) through hydrolysis catalyzed by myrosinase (MYR). The bioavailability of the different types of supplements is quite different, which in turn influences their therapeutic activities. According to the chemical form of the sulforaphane‐containing plant materials and the method of processing, the available supplements can be roughly divided into three categories. The first category consists of supplements that contain only glucoraphanin. The supplements of this type are hydrolyzed by intestinal microbiota and have low conversion efficiency and considerable interindividual variability, with a relative bioavailability of about 10% (Fahey et al. 2015; Yagishita et al. 2019). The second category of supplements is a formulated product that is enriched with exogenous myrosinase, for hydrolysis of glucoraphanin to start in the upper part of the digestive tract, with about 40% bioavailability (Fahey et al. 2019). If this type of formulated product is co‐delivered with an enteric coating, the bioavailability can be further increased to 72% under simulated intestinal conditions (Zhu, Cremonini, et al. 2025).

Third, there are the new, pre‐hydrolyzed preparations that deliver sulforaphane in its intact molecule form. They do not rely on myrosinase and gut microbes and achieve a bioavailability of approximately 70% under optimized in vitro conditions (Vanduchova et al. 2019; Fahey et al. 2015). However, this value is obtained under very controlled laboratory conditions, and in vivo bioavailability is typically much lower than such in vitro estimates. Apart from these three types of conventional supplements, another alternative is to consume fresh broccoli sprouts. Clinical comparative studies have shown that the bioavailability of fresh sprouts is three to four times higher than that of an equivalent dose of a conventional glucoraphanin supplement. However, fresh sprouts have poor stability and are much influenced by storage and cooking conditions. Therefore, formulation difference is important when using sulforaphane; taking preparations with active myrosinase or pre‐hydrolyzed sulforaphane can largely increase bioavailability.

### 3.3 In‐Depth Analysis of Biotransformation and Metabolic Pathways

Microbiota in the gut are powerful biotransformers that convert glucoraphanin (the precursor) and SFN into bioactive isothiocyanate metabolites (Khan et al. 2023). Gut microflora have an important role in the transformation process and, as such, they can augment the bioavailability and bioactivity of SFN (Khan et al. 2023). Also, SFN activates the nuclear factor erythroid 2‐related factor 2 (Nrf2) signaling pathway and causes nuclear translocation of Nrf2, which binds to the antioxidant response element (ARE). Activation results in increased expression of phase II detoxifying enzymes and antioxidant defenses such as glutathione (GSH) and superoxide dismutase (SOD), boosting cellular antioxidant mechanisms and metabolite clearance (Bose et al. 2020). The presence of SFN in skeletal muscle tissue plays an important role in the therapeutic effects of SFN on skeletal muscle disorders. It is still not clear exactly how SFN regulates muscle metabolism, reduces inflammation, and modulates muscle repair processes, and further research is needed to understand this.

## 4 Mechanisms of Action of SFN in Skeletal Muscle

### 4.1 Precise Regulation of Skeletal Muscle Protein Metabolism by Sulforaphane

Sulforaphane modulates skeletal muscle protein metabolism by increasing protein synthesis and suppressing protein degradation. Sulforaphane modulates muscle protein homeostasis via the Akt/Foxo1 axis and mTOR‐associated signaling; mTOR itself is a major regulator of muscle protein synthesis (Son et al. 2017). mTOR induces ribosome biosynthesis, translation initiation, and the capacity for protein synthesis. These effects of sulforaphane on skeletal muscle protein anabolic and catabolic pathways could protect the skeletal muscle mass in various populations at risk for skeletal muscle atrophy, such as the aging population and athletes (Wu et al. 2023; Sato et al. 2021).

### 4.2 Antioxidant Effects and Skeletal Muscle Injury Repair of Sulforaphane: Acute vs. Long‐Term Responses

Sulforaphane has potent antioxidant effects and reduces stress‐induced damage in skeletal muscle. Its predominant action is the induction of the Nrf2 pathway to increase the expression of antioxidant enzymes and the antioxidant capacity of cells. Sulforaphane stimulates the production of intracellular glutathione and protects cells from oxidative stress‐induced damage (Bose et al. 2020). Sulforaphane also acts on the redox‐related energy sensors and stress regulators (AMPK and FOXO3a) and can help to maintain homeostasis across multiple tissues (Li et al. 2020). On the other hand, repeated long‐term sulforaphane exposure results in sustained induction of Nrf2‐dependent genes and chronic adaptation of antioxidant defenses. For example, Flockhart (Flockhart et al. 2023) reported that 1‐week supplementation with glucosinolate‐rich broccoli sprouts with intense training could significantly increase the abundance of skeletal muscle Nrf2 protein and decrease oxidative stress biomarkers (carbonyl content and myeloperoxidase) in healthy subjects. Similarly, in another study, Komine (Komine et al. 2021) found that 2 weeks of 30 mg per 3 tablets sulforaphane supplementation increased mRNA expression of NQO1, the target of Nrf2, in PBMC, and also reduced delayed‐onset muscle soreness (DOMS) following eccentric exercise‐induced muscle damage. Altogether, these various effects contribute to the protection of exercise‐induced organ damage, with potential relevance to skeletal muscle homeostasis (Ruhee et al. 2020).

### 4.3 Fine Regulation of Skeletal Muscle Inflammatory Response by Sulforaphane: Acute Inhibition and Long‐Term Pro‐Repair Effects

Sulforaphane also critically regulates the inflammation process in skeletal muscle. In the acute phase, sulforaphane suppresses activation of major inflammatory pathways and hence downregulates production of cytokines and chemokines through inhibition of the NF‐κB signaling pathway, so as to restrain excessive inflammation (Treasure et al. 2023). Such suppression is necessary for muscle injury repair as a controlled inflammation programme can help muscle tissue regeneration, whereas excessive inflammation aggravates muscle damage (Sato et al. 2021). Conversely, continuous or repeated administration of sulforaphane shifts the balance of macrophage polarization toward an anti‐inflammatory, pro‐repair M2 phenotype, and thus further enhances the muscle repair process (Sato et al. 2021; Bahiraii et al. 2022). In addition, a 2‐week high‐glucoraphanin broccoli powder supplementation trial in non‐trained individuals showed no significant improvement in exercise‐induced oxidative stress markers or muscle power recovery despite confirmed systemic absorption of sulforaphane metabolites (Cesanelli, Venckunas, et al. 2026), suggesting that short‐term dietary exposure may be insufficient to produce measurable benefits under mild oxidative challenge and that effects are likely protocol‐dependent, injury‐severity‐dependent, and modulated by individual bioavailability. Overall, these results point to the possible usefulness of sulforaphane for chronic muscle inflammation, and more clinical work is clearly needed to demonstrate that.

### 4.4 Precise Regulation of Skeletal Muscle Metabolic Reprogramming by Sulforaphane

**FIGURE 1:**
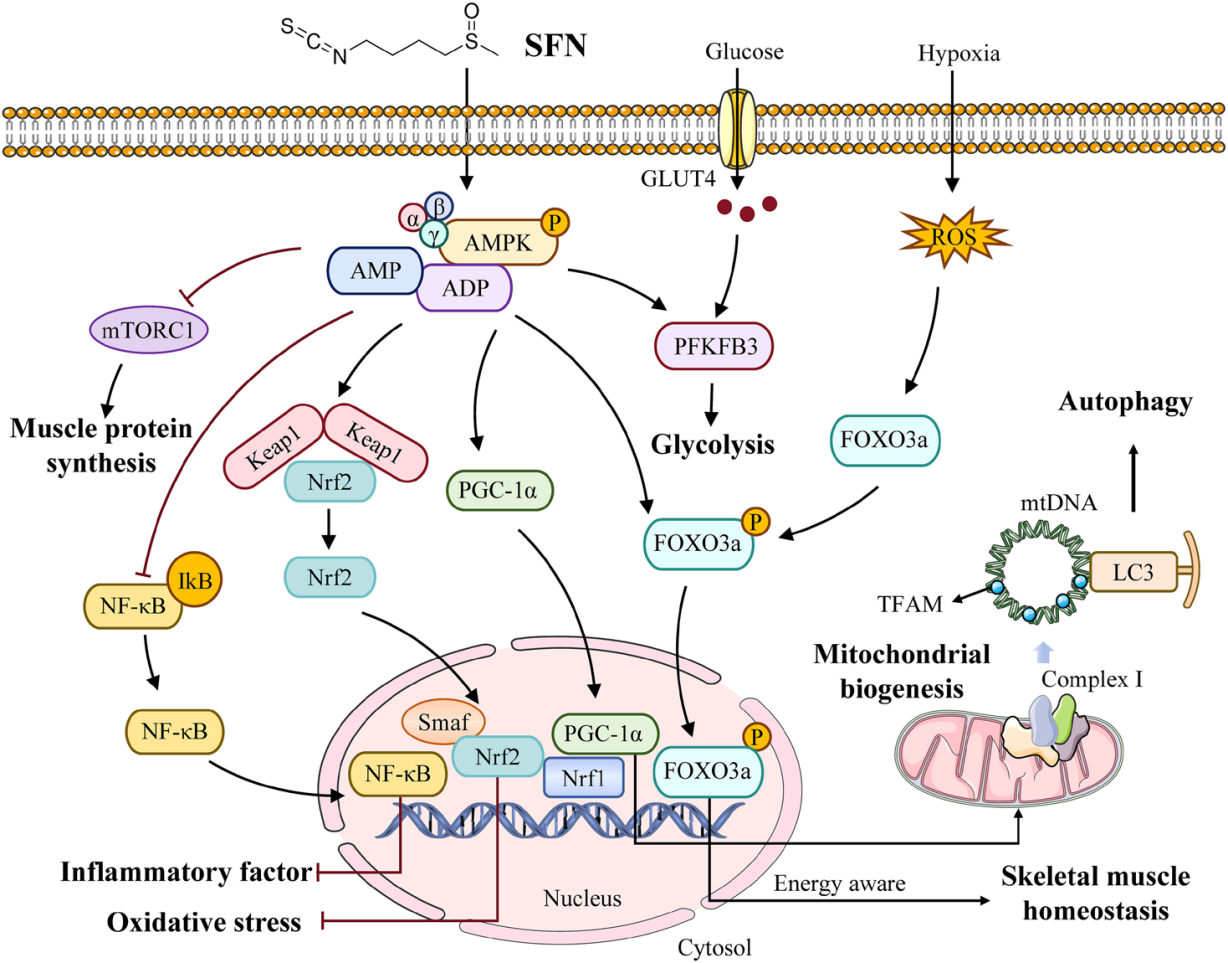
The mechanism of sulforaphane (SFN) in skeletal muscle. Schematic illustration of sulforaphane (SFN) signaling network in skeletal muscle. SFN simultaneously modulates four interconnected pathways. (1) SFN activates mTORC1 to promote protein synthesis and regulates FOXO3a to balance autophagy and protein degradation. (2) SFN modifies Keap1 to release Nrf2, which translocates to the nucleus and upregulates antioxidant enzymes via ARE; Nrf2 also cross‐talks with FOXO3a and NF‐κB. (3) SFN inhibits NF‐κB signaling (via IκB) and suppresses inflammatory cytokine production. (4) SFN activates AMPK, which enhances glycolysis through GLUT4/PFKFB3 and induces mitochondrial biogenesis via PGC‐1α–TFAM. Subcellular localization (cytosol vs. nucleus) is indicated. Arrows indicate activation; T‐bars indicate inhibition. ARE, antioxidant response element; FOXO3a, forkhead box O3a; GLUT4, glucose transporter type 4; IκB, inhibitor of NF‐κB; Keap1, Kelch‐like ECH‐associated protein 1; LC3, microtubule‐associated protein 1A/1B‐light chain 3; mTORC1, mechanistic target of rapamycin complex 1; NF‐κB, nuclear factor kappa‐B; Nrf2, nuclear factor erythroid 2‐related factor 2; PFKFB3, 6‐phosphofructo‐2‐kinase/fructose‐2,6‐bisphosphatase 3; PGC‐1α, peroxisome proliferator‐activated receptor gamma coactivator 1‐alpha; TFAM, mitochondrial transcription factor A.

The benefit of sulforaphane on skeletal muscle metabolic reprogramming mainly comes from a change in energy metabolism. Studies in immune cells show that sulforaphane induces a shift in substrate utilization from fatty acid oxidation to glycolysis (a Warburg‐like phenotype) (Bahiraii et al. 2022); a similar metabolic remodeling has been proposed in skeletal muscle but requires direct experimental validation. Sulforaphane can modulate metabolic signaling pathways relevant to energy homeostasis and enhance skeletal muscle cell function (Zhang et al. 2024). These metabolic effects point to the future therapeutic significance of sulforaphane on metabolic myopathies and diabetic myopathy. Overall, SFN can precisely regulate skeletal muscle metabolic reprogramming by coordinating glycolysis and mitochondrial biogenesis activation via AMPK‐mediated pathways to provide a theoretical basis for using SFN in metabolic skeletal muscle disease (Figure 1). Table 1 briefly summarizes the experimental basis for the four core pathways in which SFN regulates skeletal muscle function.

**TABLE 1:** Summary of key experimental evidence for the four core pathways of Sulforaphane (SFN) regulating skeletal muscle function.

Core pathway	Experimental model	SFN intervention	Key targets/indicators (with specific quantitative changes)	Pathway regulatory effects	Key limitations	References
Protein homeostasis regulation	C2C12 myotubes (1 μmol/L dexamethasone‐induced atrophy)	5 μmol/L, in vitro incubation for 24 h	Myostatin and Atrogin‐1 mRNA decreased by 30%; MyoD protein expression increased by 20%; p‐Akt (Ser473) increased by 2‐fold; p‐Akt (Thr308) increased by 3‐fold; p‐Foxo1 increased by 3‐fold; myotube diameter increased by 1.2‐fold	Promotes skeletal muscle protein synthesis, inhibits ubiquitin‐proteasome system‐mediated myoprotein degradation, and alleviates disuse atrophy	In vitro only; no in vivo validation; single SFN concentration tested	(Son et al. 2017)
Oxidative stress defense	C57BL/6 mice (2‐month young and 21–22‐month old)	2 mg/kg/d, oral administration via diet for 12 weeks	ETC Complex I activity increased by 30% in aged mice; Nrf2‐ARE‐binding activity increased by 2‐fold in aged mice; protein abundance of Nrf2 and its target genes (Sod1, Cat) restored to near‐young levels	Activates Nrf2 antioxidant pathway, enhances skeletal muscle antioxidant capacity and glutathione synthesis, improves mitochondrial function	Animal model only; human translation not validated; small sample size; single dose regimen	(Bose et al. 2020)
Inflammatory network remodeling	C57BL/6J mice (muscle injury model); RAW264.7 macrophages	In vitro: 25–200 μmol/L, incubation for 0.5–1 h; In vivo: 2 mg/kg i.p.	IL‐1β secretion reduced by 4‐fold; macrophage infiltration decreased by 50%; M2 macrophage polarization enhanced	Inhibits excessive inflammation via NF‐κB pathway, promotes M2 macrophage polarization to facilitate muscle repair	Mixed in vitro and acute animal models; limited chronic supplementation data; small animal group sizes	(Treasure et al. 2023; Bahiraii et al. 2022)
Metabolic phenotype reprogramming	C57BL/6J mice (high‐fat diet‐induced diabetic myopathy)	2 mg/kg/d, oral gavage 5 days/week for 3 months	PGC‐1α protein level increased by 2‐fold; mitochondrial biogenesis markers up‐regulated by 1.5–2‐fold; insulin sensitivity improved; p‐AMPK/AMPK ratio increased by 2‐fold	Activates AMPK/PGC‐1α axis, promotes mitochondrial biogenesis, improves skeletal muscle insulin sensitivity and energy metabolism	Animal model only; no human clinical data; long‐term diet intervention interferes with effect attribution	(Li et al. 2020)

*Note:* For the inflammatory network remodeling row, NF‐κB inhibition and M2 macrophage polarization evidence are summarized in review (Treasure et al. 2023); IL‐1β secretion quantitative data are derived from in vitro macrophage study (Bahiraii et al. 2022).

### 4.5 Critical Appraisal of SFN'S Mechanistic Complexity and Limitations

A balanced appraisal of SFN also needs to take into account the pathway complexities and inconsistent results. First, the four main pathways (mTOR, Nrf2, NF‐κB, AMPK) do not work in isolation. Excessive mTOR activity depresses insulin sensitivity and suppresses autophagy; excessive Nrf2 signaling undermines low‐level oxidative signals needed for adaptive muscle remodeling; complete inhibition of NF‐κB signaling is counter‐productive because NF‐κB is required for satellite cell proliferation; and activation of AMPK by SFN varies across tissue types and experimental models (Li et al. 2020; Zhang et al. 2024). Second, not all studies replicated an SFN‐mediated upregulation of Nrf2 target genes. A randomized COPD trial showed that oral SFN (25 and 150 μmol) for 4 weeks did not increase NQO1 or HO‐1 despite appropriate absorption (Wise et al. 2016), and a crossover trial in healthy human volunteers showed no effect of SFN on markers of metabolic stress after caloric challenge (van Steenwijk et al. 2023).

These differences probably reflect heterogeneity of formulation, bioavailability, dose, species, muscle fiber type and treatment length. Third, SFN has hormetic and non‐linear dose responses: low doses induce a protective adaptation and high doses induce oxidative stress, disruption of mitochondrial complexes or alkylation of thiol proteins other than Keap1. Most preclinical studies report a single dose (such as 2 mg·kg^−1^·d^−1^ in mice), and dose–response information is limited. Finally, as an electrophilic isothiocyanate, SFN covalently targets cysteine residues in many of its targets other than Keap1, inhibiting redox‐sensitive transcription factors (e.g., activating protein 1 [AP‐1]), perturbing mitochondrial electron transport and blocking beneficial inflammatory signaling. Proteome‐wide profiling is required to discover the complete target profile of SFN in skeletal muscle. Taken together, SFN effects are context‐dependent and more careful and rigorous studies are needed before confident clinical translation is possible.

## 5 Application of Sulforaphane in Different Skeletal Muscle Diseases

### 5.1 Sulforaphane and ICU‐AW

ICU‐acquired weakness (ICU‐AW) is a common complication of critical illness, affecting a substantial proportion of ICU patients and characterized by muscle atrophy, impaired function, and prolonged recovery (Lad et al. 2020; Schefold et al. 2020). Sulforaphane may be effective for ICU‐AW through several mechanisms. First, sulforaphane modulates muscle protein homeostasis and protects against disuse muscle atrophy (Son et al. 2017). Second, sulforaphane may attenuate muscle damage through NF‐κB pathway inhibition (Nallasamy et al. 2014); however, this mechanistic inference is primarily derived from vascular endothelial cell studies, and direct evidence in skeletal muscle tissue remains limited. Third, sulforaphane enhances cellular antioxidant defenses by activation of Nrf2 and thus prevents oxidative stress‐induced damage (Komine et al. 2021). However, direct evidence for SFN in validated ICU‐AW models remains limited; most supportive data are derived from non‐ICU contexts such as exercise‐induced muscle damage or disuse atrophy models (Son et al. 2017; Komine et al. 2021), and extrapolation to critically ill patients requires caution.

A randomized controlled study of patients with COPD did not show effects of 4 weeks' treatment with SFN by mouth (doses of 25 μmol and 150 μmol) on expression of Nrf2 target genes or inflammatory marker concentrations, even though adequate oral absorption was demonstrated (Wise et al. 2016). A review of 84 registered clinical studies with SFN showed that about half were unpublished, and among those published, outcomes have been inconsistent—particularly for respiratory diseases in which SFN had no effect and in hypertension in which no benefit was observed (Saito et al. 2025). These results strongly indicate that SFN is context dependent and perhaps not globally reproducible. Therefore, although sulforaphane has some promise in the care of critically ill patients, a more realistic view is its possible role as an adjuvant therapy to an existing clinical strategy, not as an independent agent for ICU‐AW.

### 5.2 Sulforaphane and Exercise‐Induced Muscle Damage: Prevention and Repair

Exercise‐induced muscle damage is a concern in the sports and fitness community. Emerging evidence suggests that sulforaphane could be useful for muscle damage in the sports and fitness world from different aspects. Moreover, sulforaphane may support muscle repair and regeneration via its antioxidant and anti‐inflammatory properties (Bose et al. 2020). Thus, sulforaphane administration prior to exercise could reduce the incidence of muscle injury, and administration after exercise could facilitate muscle injury repair. Meanwhile, sulforaphane can also promote antioxidant function by activating the Nrf2/HO‐1 signal pathway to protect against exercise‐induced organ damage in preclinical models (Ruhee et al. 2020). Its underlying dual‐phase mechanism can meet the current needs of sports medicine, indicating that sulforaphane can be used as a nutritional supplement to support muscle resilience and recovery in athletes.

Regarding exercise performance, Flockhart (Flockhart et al. 2023) showed that 7 days of intake of 2 bottles of glucosinolate‐rich broccoli sprout juice per day enhanced performance and lowered oxidative stress during high‐intensity training. In contrast, Cesanelli (Cesanelli, Thomas, et al. 2026) showed that 2 weeks of high‐glucoraphanin broccoli powder supplementation did not influence the recovery of isometric and isokinetic peak torque, muscle soreness, or creatine kinase levels following elbow flexor eccentric exercise in a double‐blind, placebo‐controlled crossover trial. These different findings (improved performance and low oxidative stress in one study versus no effect on exercise recovery in other studies) indicate that the effectiveness of sulforaphane may depend on exercise mode, duration of intake, the individual's baseline oxidative stress status, and interindividual conversion capacity. Therefore, both acute and chronic effects of sulforaphane should be taken into consideration to understand the role of sulforaphane in exercise muscle damage and repair.

### 5.3 Sulforaphane and Diabetic Myopathy: Addressing Metabolic Dysfunction

Diabetic myopathy is a common complication of diabetes mellitus and is characterized primarily by muscle atrophy and muscle metabolic disturbance (Bassi‐Dibai et al. 2022). Sulforaphane modulates lipid and protein metabolism‐related signaling pathways in skeletal muscle (Zhang et al. 2024), so it may offer protective effects against diabetic muscle injury. Most evidence supporting these protective effects comes from in vitro and animal studies, and direct evidence in human skeletal muscle remains scarce. A caloric challenge trial by van Steenwijk et al. found no effect of SFN on biomarkers of metabolic stress (van Steenwijk et al. 2023). These findings highlight the context‐dependent nature of SFN efficacy and underscore the need for dose optimization and careful patient stratification in future trials.

### 5.4 Sulforaphane and Respiratory Muscle Dysfunction in COPD, Diabetes and Obesity

Respiratory muscle dysfunction is a frequent yet often unnoticed complication of COPD, diabetes mellitus, and obesity. The main inspiratory muscle, the diaphragm, is mostly involved; accessory muscles (for instance, external intercostal and sternocleidomastoid muscles) are recruited when overloaded.

Across these disease states, the causes of diaphragm dysfunction differ in detail but share mechanical overload and metabolic stress as common mechanistic themes. In obesity, increased intra‐abdominal adipose tissue raises intra‐abdominal pressure and can impose mechanical overload on the diaphragm, for example. Mechanical overload can also lead to myofiber damage and changes to sarcomere arrangement, reduced contractility efficiency, and progressive loss of capacity to compensate for the overload without treatment (Cesanelli et al. 2024). In COPD, chronic hyperinflation flattens the diaphragm and decreases the diaphragm operating length and reduces the force‐generating capacity of the diaphragm, all potentially accompanied by fiber type shifting and diaphragmatic atrophy. In diabetes, insulin resistance, AGE accumulation, and systemic oxidative stress can aggravate diaphragm contractility impairment and diaphragm wasting. Classic counteracting strategies include weight loss (or bariatric surgery) for obesity, inspiratory muscle training (IMT), and continuous positive airway pressure (CPAP) to unload the respiratory system, bronchodilators for COPD, and strict glycemic control for diabetes. Each of them provides partial benefit but with limitations: weight loss is slow and difficult to sustain, IMT requires very good patient compliance, and drug therapy does not address oxidative and inflammatory damage in the diaphragm.

Sulforaphane (SFN) has recently been proposed as a complementary strategy to address these gaps, although evidence in this direction is at present preliminary. As a known Nrf2 inducer, SFN may lower oxidative stress and inflammation in skeletal muscle, including the diaphragm. By maintaining the mitochondrial complex I activity and increasing bioenergetics, SFN may help protect the diaphragm myofibers from overload‐induced injury. Direct evidence in the diaphragm is sparse and most of the data are from other non‐respiratory muscle tissues or in vitro models. SFN possesses favorable safety profiles, yet its protective effects targeting respiratory muscles lack robust clinical validation; adjunct application alongside standard therapy remains an exploratory research direction.

### 5.5 Sulforaphane and Sarcopenia: Targeting Age‐Related Muscle Wasting

Sarcopenia, an aging‐related condition, is characterized by progressive loss of muscle mass and strength (Cruz‐Jentoft et al. 2019). Sulforaphane has been studied as a possible treatment of sarcopenia, in particular for its potential role in satellite cell activation and mTOR signaling (Son et al. 2017). Diet‐supplementation with sulforaphane may improve the limb strength capacity of aged mice. Furthermore, the improved muscle function in aged mice may be partially attributed to enhanced satellite cell activity, which requires dedicated experimental validation. Also, as an Nrf2 activator, sulforaphane improves mitochondrial bioenergetics in aged C2C12 myotubes and ameliorates muscle atrophy in aged wild‐type mice (Yan et al. 2022). Therefore, intervention for sarcopenia should consider a nutritional approach, physical activity and selective sulforaphane supplementation together. Unlike monotherapies, such a multimodal strategy takes advantage of the mutually beneficial effects of dietary support and physical activity (Ruhee et al. 2020; Zeng et al. 2024; Parkington et al. 2003), and is a holistic approach to maintenance of musculoskeletal health in the elderly. This holistic approach is useful not only to maintain muscle quality but also to improve the quality of life in older individuals.

## 6 Clinical Translation Challenges and Countermoves for Sulforaphane

### 6.1 Profound Limitations in Mechanistic Elucidation

It is not yet clear what the mechanisms of sulforaphane activity are in skeletal muscle disorders, particularly in regard to the differential effect of sulforaphane on fast‐twitch and slow‐twitch muscle fibers. Electrical stimulation studies in rat skeletal muscle have shown striking differences in the level of mTOR phosphorylation in type IIA (fast‐twitch) fibers versus type I (slow‐twitch) fibers (Parkington et al. 2003) that may explain the drastic differences in the responsiveness of fast and slow muscle fibers to sulforaphane. In respiratory muscle‐specific mechanisms studies, high levels of Nrf2 expression were shown in the diaphragm compared with the quadriceps femoris (Hashimoto et al. 2024), indicating a tissue‐specific effect of sulforaphane. The long‐term effects of sulforaphane on the nuclear domain organization of myocytes are also still unknown—something that will have an important influence on muscle function and capacity for muscle regeneration. To address these mechanistic uncertainties, single cell RNA sequencing would be helpful to understand gene expression patterns of myofiber subtypes and their responses to sulforaphane.

### 6.2 Individual Response Heterogeneity and Precision Dosing

Interindividual variability in the response to sulforaphane is substantial, driven by factors including genetic differences (including a mutation at the gene locus for Nrf2 rs6721961). Strategies are needed to address such problems; precision dosing is one, in which TDM, 16S rRNA profiling of the microbiome, and individual genotyping are integrated to develop machine‐learning‐driven dosing models that might allow for individualized and adaptive dose optimization. Such models quantitatively explore impacts of influencing factors (e.g., genetic polymorphisms (such as Nrf2, Keap1 and GSTM1), gut microbiota composition (particularly myrosinase‐producing bacteria), hepatic and renal functions) on sulforaphane metabolism and response. Such interventions move away from one‐size‐fits‐all approaches and ensure that each individual patient receives personalized dosing schedules relevant to their individual physiology and make‐up.

### 6.3 Research Design Optimization and Evidence Enhancement

For clinical trials of sulforaphane, study design needs optimization, in particular, sulforaphane for critically ill patients. Future trials for sulforaphane should use “diaphragm contraction recovery time” (Luo et al. 2025) and “successful extubation rate” (Fan et al. 2017) as primary endpoints in the ICU‐AW trials and in a multicenter randomized controlled trial, which should strictly control for confounders (e.g., the choice of sedative (Devlin et al. 2018) and nutrition supplement (Singer et al. 2023)). Research plans need to be formulated according to particular patient types. For example, the study for ICU‐AW should strictly control confounders such as sedation and ventilator time, using muscle ultrasound and assessment of respiratory muscle function (Yao et al. 2024). Studies of sarcopenia need to take both sarcopenic obesity and the frailty phenotype into account and use “risk of falling” as a primary endpoint (Ozturk et al. 2018). For the diabetic myopathy, analyses for the course of the disease need to be adjusted for the levels of blood glucose and insulin (Sugimoto et al. 2021). Mixed intervention strategies (e.g., combining SFN with exercise or nutritional supplementation) should be explored. Future work should include co‐administration of sulforaphane with well‐established anabolic stimuli (e.g., resistance exercise, leucine supplementation or β‐hydroxy‐β‐methylbutyrate [HMB]) in a manner where safety and ethical issues are properly considered. Optimizing these studies will allow us to quickly translate promising preclinical findings to clinical studies, identify crucial technical bottlenecks, develop feasible solutions and improve the translational relevance and scientific impact of the field.

### 6.4 Regulatory and Safety Considerations for Sulforaphane

Another feature of sulforaphane is its well‐established toxicological profile, which has an LD_50_ of approximately 213 mg/kg in mice after intraperitoneal administration (Mangla et al. 2021). Glucoraphanin sourced from broccoli seeds received FDA GRAS notification (No. 496) in 2013, with a ‘no objection’ letter issued in 2016 and can thus be considered a safe food ingredient. Clinically, sulforaphane formulations (e.g., Avmacol) are being investigated in investigator‐initiated Phase II/III clinical trials reported in the literature for conditions such as type 2 diabetes (NCT02801448), chronic kidney disease (NCT05797506), and autism spectrum disorder (ASD) (NCT02561481). These reports indicate that although there are still translational issues to be overcome, the safety profile of sulforaphane is well established, and preliminary clinical data support further exploration of sulforaphane for skeletal muscle disorders. Future research will have to further evaluate long‐term safety and the occurrence of rare adverse effects to guarantee patient safety and clinical use.

## 7 Future Research Prospects

### 7.1 Mechanism Analysis Driven by Advanced Technologies

Recent development of biotechnology, such as organ‐on‐a‐chip models, has opened up new opportunities for us to study the role of sulforaphane in skeletal muscle diseases. A 3D co‐culture model comprising diaphragmatic myocytes, endothelial cells, and immune cells can be established to mimic the ICU‐AW microenvironment and monitor the time course of sulforaphane intervention effect on the atrophy of respiratory muscle in real time. Such a model can reflect in vivo physiology better and facilitate a detailed study on intercellular interaction. Isotope‐assisted metabolic flux analysis (Moiz et al. 2022), combined with skeletal muscle lipid mass spectrometry (Moreno‐Torres et al. 2019), can be applied to dissect SFN‐mediated metabolic shifts in diabetic myopathy models. Our integrated approach allows us to decipher the action of sulforaphane under different metabolic conditions and provides a solid basis for future clinical applications (Moiz et al. 2022).

### 7.2 Precision Medicine‐Driven Translational Innovation

**FIGURE 2:**
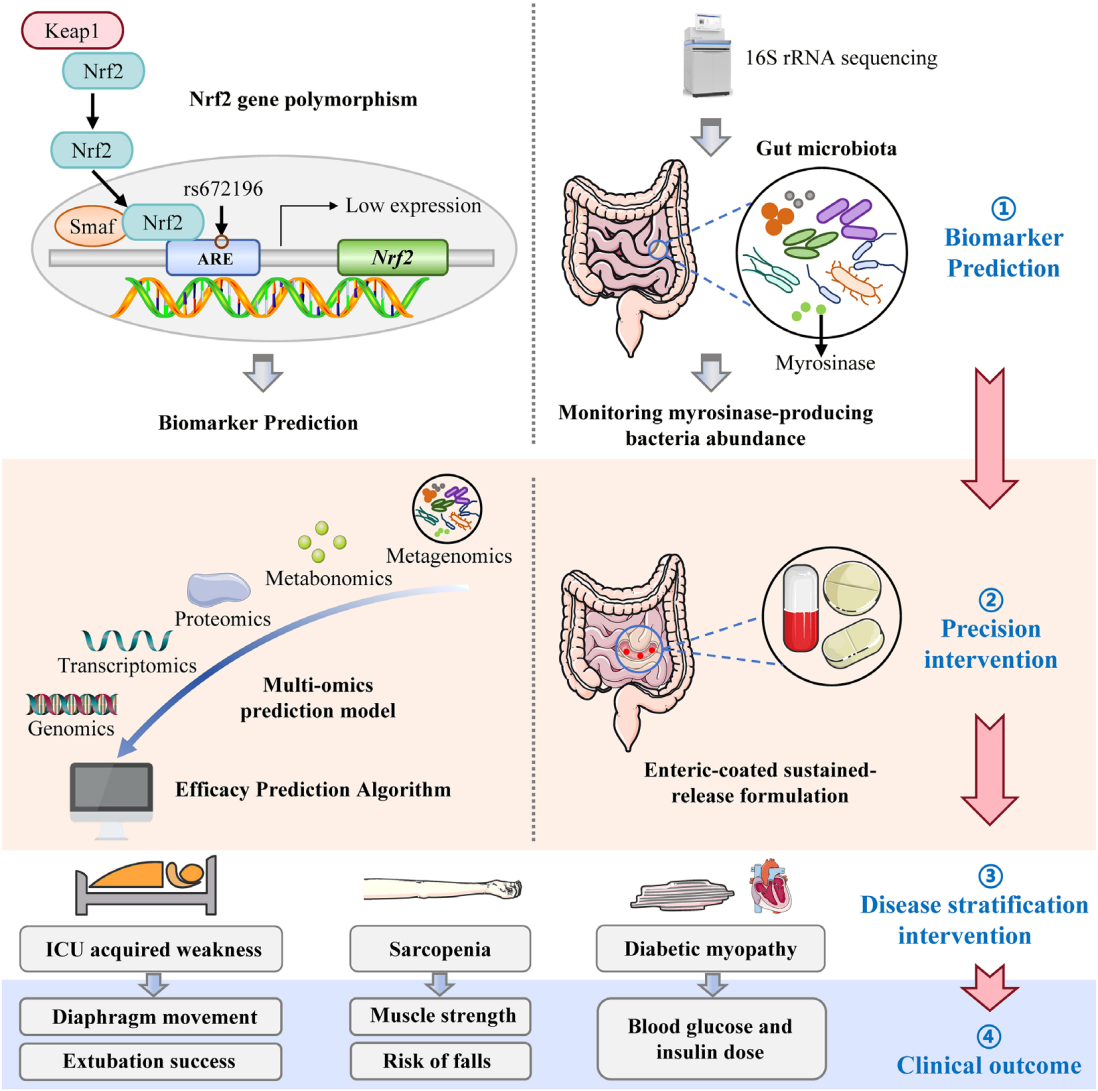
Framework diagram of precision medicine implementation of SFN in skeletal muscle diseases: Conceptual framework for precision medicine implementation of sulforaphane (SFN) in skeletal muscle diseases. The framework comprises four interconnected levels: (1) Biomarker stratification: Biomarkers include Nrf2 polymorphisms (e.g., rs6721961), Keap1 genotype, and gut microbiota composition (abundance of myrosinase‐producing bacteria), assessed via 16S rRNA sequencing and metagenomics. Nrf2–ARE binding activity serves as a functional biomarker. (2) Precision intervention: Multi‐omics integration (genomics, transcriptomics, proteomics, metabolomics, metagenomics) informs machine learning‐driven efficacy prediction algorithms and dose optimization. Smart delivery systems (e.g., enteric‐coated sustained‐release formulations) synchronize SFN release with real‐time gut microbiome activity. (3) Disease stratification: Interventions are tailored to distinct patient populations—ICU‐acquired weakness (ICU‐AW), sarcopenia, and diabetic myopathy—using disease‐relevant endpoints. (4) Clinical outcomes: Outcomes include diaphragm movement recovery time and extubation success (ICU‐AW), muscle strength and fall risk (sarcopenia), and blood glucose and insulin levels (diabetic myopathy). This framework enables individualized SFN therapy beyond one‐size‐fits‐all approaches.

Within the concept of precision medicine, multi‐omics predictive models offer exploratory strategies for individual sulforaphane interventions. While multi‐omics predictive frameworks for personalized intervention remain exploratory and require further validation, analytical tools including isotope‐assisted metabolic flux analysis (Moiz et al. 2022) provide foundational technical support for constructing individual response prediction models. Another exploratory way is to develop a targeted drug delivery system (Dong et al. 2021; Zhu, Liu, et al. 2025; Chen et al. 2020). For example, an enteric‐coated sustained‐release formulation that releases the precursor in response to gut microbiome activity can improve bioactivation efficiency in responders (Chen et al. 2020; Liu et al. 2022). A recent bibliometric study has reported that sulforaphane is the most discussed Nrf2‐activating phytochemical (Paunkov et al. 2019). Accordingly, Nrf2 activation status may be the key biomarker for predicting sulforaphane response in the context of precision nutrition. Through basic mechanistic studies, biomarker screening, and personalized intervention, it would be even more promising to achieve precision medicine of sulforaphane (Figure 2).

Unlike some previous reviews, which were focused mainly on the beneficial effects of SFN in skeletal muscle, our review has three distinct strengths. Firstly, we distinguish between acute and chronic effects of SFN supplementation from different study models. Secondly, we critically evaluate negative and inconclusive clinical trials and provide an objective assessment of the translational gap. Thirdly, we propose a research agenda (which includes biomarker stratification (Nrf2 genotype, gut microbiota myrosinase activity), dynamic SFN delivery and multi‐omics prediction models) which goes well beyond overly simplistic claims of one‐size‐fits‐all precision medicine to testable hypotheses for future trials.

### 7.3 Public Health and Policy Transformation

For public health and clinical nutrition research, the clinical trials reviewed above are largely negative or inconclusive, so recommending sulforaphane‐enriched enteral nutrition for ICU patients would be premature. Sepsis is one of the most frequent causes of death in the ICU and a major driver of ICU‐AW. Sulforaphane may protect against muscle damage through its well‐characterized antioxidant and anti‐inflammatory pathways (Treasure et al. 2023). From a dietary perspective, broccoli sprouts can be used to prepare functional foods with improved nutritional value. Dietary supplementation with sulforaphane has been shown to attenuate age‐related decline in skeletal muscle function (Bose et al. 2020; Zeng et al. 2024). These actions may help translate sulforaphane research into public health policy and clinical practice and could benefit a broad patient population.

## 8 Conclusion

Sulforaphane is a naturally occurring isothiocyanate derived from cruciferous vegetables and is one of the multifunctional cytoprotective agents for skeletal muscle. Its pleiotropic effects on protein homeostasis, redox balance, inflammation, and metabolism show preliminary therapeutic promise and suggest that sulforaphane may complement other therapies in musculoskeletal diseases. Preclinical and early clinical evidence supports a potential adjunctive role of SFN in the management of ICU‐AW, age‐related sarcopenia, and diabetic myopathy, complementing existing therapeutic strategies.

From a translational perspective, the evidence reviewed here supports three distinct application tiers with different levels of evidence: (i) dietary intake of cruciferous vegetables as part of a healthy diet, supported by epidemiological and associative data; (ii) functional foods enriched with glucoraphanin or myrosinase, requiring product‐specific bioavailability validation; and (iii) standardized nutraceutical supplements delivering defined doses of SFN or its precursors, for which preclinical and early‐phase clinical data are most abundant but still insufficient to recommend routine therapeutic use. These tiers must not be conflated when interpreting evidence strength or making clinical recommendations.

Despite current evidence, there are gaps between mechanistic evidence and clinical translation of SFN. First, although the mechanisms of SFN have been partially disclosed, heterogeneity in the experimental setting, sample size, statistical power, and outcome parameters among different studies may all influence inconsistent results. Second, poor bioavailability, low solubility, low stability, and absence of formulation standardization of SFN all pose limitations for its development and clinical use. Developing new drug carriers is a direction for future research. Existing evidence inspires the development of SFN for clinical application in critically ill patients, and its safety and efficacy need to be proven by a high‐quality Phase III clinical trial. Furthermore, the dose–response relationship of SFN remains poorly characterized, and the effective therapeutic range in humans has not been established; doses used in some animal studies exceed clinically achievable exposures (Fahey et al. 2015; Yagishita et al. 2019).

Thus, better dosing models will have to be developed to maximize personalized treatment and to support the clinical development of SFN as a nutraceutical intervention. Future progress will also depend on bridging and combining different rational drug design concepts with interdisciplinary research. Bridging systems biology, pharmacokinetics, and good clinical trial design will assist in refining experimental design and catalyze translational innovation. In general, despite being in its early stages, clinical translation of SFN toward skeletal muscle disorders will be informed by larger, well‐designed clinical studies that address key knowledge gaps in muscle protection research. With further research advancements, sulforaphane could be a very helpful option for treating skeletal muscle‐related diseases. However, clinical use of SFN is exploratory at the present stage until robust and adequate phase III trial results, along with advanced criteria for patient selection, are available.

## Author Contributions

**Juan Chen:** conceptualization, methodology, software. **Guoyun Zhu:** validation, supervision, data curation. **Zhongli Zhu:** investigation, validation. **Bo Huang:** conceptualization, methodology. **Jian Hu:** supervision, validation, visualization. **Junya Li:** software, data curation. **Jian Feng:** writing – review and editing, writing – original draft, conceptualization. **Fuxiang Li:** project administration, resources, supervision. **Minru Zhao:** writing – original draft, writing – review and editing, data curation.

## Funding

The authors have nothing to report.

## Disclosure

This manuscript is a narrative review and does not report findings from a primary clinical trial conducted by the authors. The trial registration numbers mentioned in the main text (e.g., NCT02801448, NCT05797506, NCT02561481) refer to previously published studies by other investigators and are cited solely for contextual discussion. AI‐assisted tools (e.g., Grammarly and Baidu Translate) were used solely for language editing and formatting, and that all scientific content was independently reviewed and verified by the authors.

## Conflicts of Interest

The authors declare no conflicts of interest.

## Data Availability

Data sharing not applicable to this article as no datasets were generated or analysed during the current study.
